# Dissecting the Meiotic Recombination Patterns in a *Brassica napus* Double Haploid Population Using 60K SNP Array

**DOI:** 10.3390/ijms24054469

**Published:** 2023-02-24

**Authors:** Shuxiang Yan, Jianjie He, Mi Tang, Bangfa Ming, Huaixin Li, Shipeng Fan, Yiyi Xiong, Hongbo Chao, Libin Zhang, Aihua Wang, Maoteng Li

**Affiliations:** 1 Department of Biotechnology, College of Life Science and Technology, Huazhong University of Science and Technology, Wuhan 430074, China; 2 School of Agricultural Sciences, Zhengzhou University, Zhengzhou 450001, China; 3 Key Laboratory of Molecular Biophysics of the Ministry of Education, Wuhan 430074, China; 4 Wuhan Vegetable Research Institute, Wuhan Academy of Agriculture Science, Wuhan 430345, China

**Keywords:** *Brassica* 60K array, *Brassica napus*, crossover, recombination, CO frequency

## Abstract

Meiotic recombination not only maintains the stability of the chromosome structure but also creates genetic variations for adapting to changeable environments. A better understanding of the mechanism of crossover (CO) patterns at the population level is useful for crop improvement. However, there are limited cost-effective and universal methods to detect the recombination frequency at the population level in *Brassica napus*. Here, the *Brassica* 60K Illumina Infinium SNP array (*Brassica* 60K array) was used to systematically study the recombination landscape in a double haploid (DH) population of *B. napus*. It was found that COs were unevenly distributed across the whole genome, and a higher frequency of COs existed at the distal ends of each chromosome. A considerable number of genes (more than 30%) in the CO hot regions were associated with plant defense and regulation. In most tissues, the average gene expression level in the hot regions (CO frequency of greater than 2 cM/Mb) was significantly higher than that in the regions with a CO frequency of less than 1 cM/Mb. In addition, a bin map was constructed with 1995 recombination bins. For seed oil content, Bin 1131 to 1134, Bin 1308 to 1311, Bin 1864 to 1869, and Bin 2184 to 2230 were identified on chromosomes A08, A09, C03, and C06, respectively, which could explain 8.5%, 17.3%, 8.6%, and 3.9% of the phenotypic variation. These results could not only deepen our understanding of meiotic recombination in *B. napus* at the population level, and provide useful information for rapeseed breeding in the future, but also provided a reference for studying CO frequency in other species.

## 1. Introduction

Meiotic recombination is a basic genetic phenomenon in most eukaryotes, which generates genetic variations by a reciprocal exchange between the parental homologous chromosomes and leads to the production of new alleles. During meiosis I, meiotic recombination is triggered by DNA double-stranded breaks (DSBs) [1], and rigorous DSB repair is then carried out by a series of recombinant associated proteins [2,3]. In this process, a four-way DNA intermediate Holliday junction mediates the homologous sequence repair [4]. DSB repair will result in either crossovers (COs) between the sister chromatids or non-crossovers (NCOs) between the non-sister chromatids [5]. COs are classified into two types, type I and type II. Type I COs are interference-sensitive, and their production relies on the ZMM (Zip1-4, Mer3, and Msh4-5 proteins) pathway [6], whereas type II COs are interference-insensitive and their formation depends on an alternative pathway [7]. Generally, COs are associated with the exchange of long DNA fragments between the homologous chromosomes [8]. In plants, the majority of COs belong to type I, which is a form of obligatory CO [9].

The detection of meiotic recombination profiles in specific organisms should be used when segregating populations. It was found that COs varied according to the different species, and sexual differences in the same organism could also affect the production of COs. In human beings, higher recombination rates in females than those in males were found across the entire genome, and sex-specific CO hotspots were also detected [10]. However, the opposite scenario was found in *Arabidopsis thaliana*; CO rates in males, but not in females, were extremely high at the distal ends of each chromosome [11]. The meiotic recombination rate could be detected by means of different strategies. One method was a classical genetic technique; for example, the recombination frequency was measured in the segregating offspring by culturing species with auxotrophic traits in the *S. cerevisiae* SK1 strain [12]. The disadvantage of this method is the limitation of the number of auxotrophic markers. Another method for measuring the recombination rates often used a single locus, whereby the genomic locations of interest could be modified by relevant restriction enzymes to distinguish homologous or heterologous chromosomes [13,14]. The drawback of this method is that it only applies to one single locus. Compared to the aforementioned methods, genomic in situ hybridization (GISH) and fluorescence in situ hybridization (FISH) are effective for quantitative recombination rate inference and can achieve genome-wide CO detection, but the resolution is relatively low [15,16,17]. Besides, recombination information can also be obtained via high-resolution whole-genome sequencing technology; this method has been successfully applied to unveil the CO patterns of microspores in maize [18], while sex-specific COs were detected by comparing the different landscapes of female and male gametophyte genomes [19,20]. More recently, a Crystal Digital PCR^TM^-based genotyping assay has been conducted to detect the meiotic recombination rate of pollen nuclei in barley [21]. However, this method required fluorescent markers, which can only quantify the recombination rate of the defined chromosome intervals.

*B. napus* (AACC, 2*n* = 38) was derived from the hybridization between *B. rapa* (AA, 2*n* = 20) and *B. oleracea* (CC, 2*n* = 18) about 7500 years ago [22]. High homology exists between the A and C subgenomes as they shared the same ancestor [23]. Here, the genotyping of a DH population in *B. napus* was obtained by using the *Brassica* 60K array, which was further used for the detection of the meiotic recombination landscape. Finally, a high-resolution recombination map was constructed using the screened single nucleotide polymorphisms (SNPs). The present results showed that the COs were unevenly distributed on the entire genome, with high density at the distal ends of each chromosome; bin intervals for important agronomic traits, such as seed oil content (SOC), were identified.

## 2. Results

### 2.1. Genotyping the DH Population in B. napus using the Brassica 60K Array

A total of 348 DH lines were obtained from the microspore culture of the F1 hybrids between the parent plants of KenC-8 and N53-2, and it was named as KN DH population [24]. By means of the Illumina HiSCAN scanner’s scanning analysis, 52,157 SNPs were detected from the *Brassica* 60K array, all the SNP sequences were filtered by BLAST against the reference genome of Zhongshuang11 (hereafter known as ZS11) [25]. As a result, a total of 44,188 high-quality SNPs were obtained. Among them, 13,783 were the polymorphic SNPs between KenC-8 and N53-2 (Table S1 in the Supplementary Materials). It was found that these polymorphic SNPs were evenly distributed on each chromosome, with an average distance of 62 kb between two adjacent markers. Then, all the SNPs were implemented to genotype the KN DH population. The genotyping results showed that only a small proportion (less than 10%) of these plants were heterozygotes. After removing the lines with disorganized and incomprehensible genotypes, 292 out of the 348 lines were used for the subsequent analysis.

### 2.2. Bin Detection and CO Distribution

**Figure 1:**
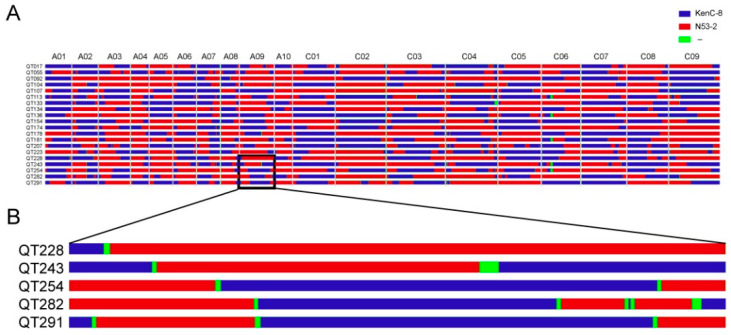
Detection of recombination bins in the KN DH population. (**A**) Recombination bins detection of 20 randomly selected lines in the KN DH population. Red intervals represent the male parent N53-2 genotype, blue intervals represent the female parent KenC-8 genotype, and green intervals indicate an undefined genotype. (**B**) Highly magnified chromosome A09 of five lines in Figure 1A.

The above genotyping results were used to search the bins of the KN DH population. Here, a haplotype interval with the same genotype across the entire population, but opposite to that of the parent, was defined as a recombination bin. In total, 2522 recombination bins were obtained (Figure 1 and Table S2 in the Supplementary Materials), which represented most of the recombination events in the KN DH population, and the average interval between two adjacent bins was 440 kb. To study the recombination landscape of the KN DH population, we first measured the replacement rate of each line. For the background of KenC-8, the replacement rate ranged from 20% (line QT213) to 77% (line QT164), and similar results were also found in the background of N53-2.

**Figure 2:**
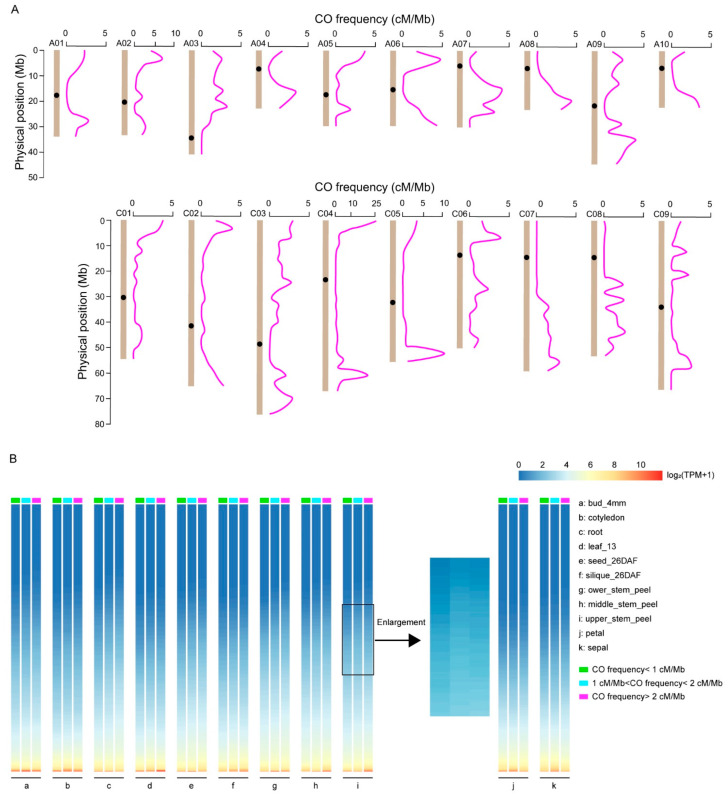
CO distribution pattern and gene expression levels in three intervals with different CO frequencies in *B. napus*. (**A**) CO frequency distribution on the 19 chromosomes of the DH population, using the ZS11 reference genome. CO frequency (cM/Mb) was calculated by genetic distance/physical length within a 3-Mb sliding window. The black dots on each chromosome represent the centromere positions. (**B**) Gene expression levels in three intervals with different CO frequencies. The genome region was divided into three intervals according to the different CO frequencies: a frequency less than 1 cM/Mb, 1 cM/Mb to 2 cM/Mb, and greater than 2 cM/Mb. A total of 11 tissues and 1000 genes randomly selected from each interval were used to analyze the gene expression level. The average gene expression level in the intervals with CO frequency > 2 cM/Mb is significantly higher than that in the intervals with CO frequency < 1 cM/Mb in the nine tissues from a to i. The enlarged diagram was used as an example to interpret the results clearly. In the rest of the two tissues (j–k), there are no significant differences in the average gene expression level among the three intervals.

The genotyping results of the KN DH population were used to detect the CO locations, and the junction of two adjacent bins was defined as a CO. Therefore, a total of 6718 COs were identified in the population (Table S3 in the Supplementary Materials). Further analysis showed that a single line contained 9 (lines QT221 and QT341) to 53 (line QT207) COs, with an average number of 23, and there were about 1.2 COs per chromosome (ranging from 0.47 to 2.8). At the genome-wide level, the CO frequency was about 1.34 cM/Mb. Specifically, the CO frequency of the A subgenome was 1.51 cM/Mb, which was higher than that of the C subgenome (1.21 cM/Mb). Chromosome A05 contained minimal CO events (178), whereas the maximal ones were found in chromosome C04 (1352) (Figure S1A in the Supplementary Materials). It was shown that the CO numbers were correlated with the chromosome length (Figure S1B in the Supplementary Materials). Further analysis revealed that the distribution of COs was uneven in the *B. napus* genome, with a trend of increase from centromeres to telomeres (Figure 2A). These results suggested that the DNA sequences that are distal from the centromeres might play an important role in maintaining genetic diversity and evolutionary processes.

**Figure 3:**
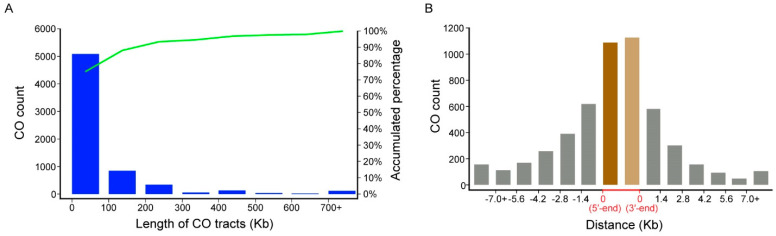
The lengths of CO and the relationships between the gene positions and CO locations. (**A**) CO tract lengths and their accumulated percentage. The scale distance is 100 Kb, and 700+ indicates a CO length greater than 700 Kb. (**B**) Potential relationships between the CO locations and gene positions. The dark brown bar indicates the number of COs close to TSS in the coding regions; the light brown bar represents the number of COs close to TTS in the coding regions; the gray bars indicate the number of COs close to TSS or TTS in the non-coding regions. The scale distance was set to 1.4 kb because the average transcript length is about 2.8 kb in the ZS11 reference genome; here, we used the median position of each CO to infer the relationships.

In order to illuminate the potential relationship between the CO distribution and the genes, their relative distribution was further analyzed. Of the 6718 COs, the intervals ranged from 7 bp to 2,790,713 bp, and around 88.4% (5938) and 75.8% (5088) were located in the 200 kb region and 100 kb region, respectively (Figure 3A). To improve the accuracy, COs with intervals shorter than 100 kb were used to infer the relationships with genes. The relative distance from the median position of each CO to its closest gene was calculated (Figure 3B). It was revealed that a high proportion of COs was distributed around the transcription start sites (TSS) and transcription termination sites (TTS) of the genes, which suggested that the genetic diversity of the offspring was frequently caused by those regulatory elements that were adjacent to the TSS and TTS.

### 2.3. CO Hot Regions and Their Genes

To better understand the distribution of COs, we defined that the region with a CO frequency greater than 2 cM/Mb within a 3 Mb window was regarded as a CO hot region. Consequently, a total of 63 CO hot regions were detected on the 19 chromosomes, and the majority of them were observed at the ends of each chromosome (Figure 2A, Table S4 in the Supplementary Materials). Besides, most of their frequencies were less than 5 cM/Mb. The fact that CO frequency could be greater than 20 cM/Mb in the regions of chromosome C04 indicated that these regions were unusually active during the meiotic recombination exchange process.

All CO hot regions occupied a 188.68 Mb physical interval, accounting for about 22% of the total genome. Through mapping these intervals to the reference genome of ZS11, a total of 25,214 genes were found to be located in these regions, which accounted for 25% of all genes. These genes were classified into 274 categories (Figure S2 and Table S5 in the Supplementary Materials), and the most significantly enriched subcategory was the ‘regulation of the salicylic acid-mediated signaling pathway’; the genes in this pathway played a critical role in the host response to microbial pathogens. Similar enriched terms for abiotic and biotic stress were also found in other subcategories, such as ‘response to heat’, ‘response to salt stress’, and ‘defense response to insect’. In addition, many subcategories that are associated with regulation were also detected, such as the ‘positive regulation of gene expression’, ‘regulation of mRNA metabolic process’, and ‘regulation of hormone metabolic process’. Among the top 100 subcategories, more than 30% of them were related to defense and regulation, which indicated that the genes in these CO hot regions were closely related to plant defense and gene regulation. Meanwhile, we also discovered that the genes classified as defense and regulation in the A subgenome were about twice that in the C subgenome, which might be induced by the hybridization and selection in the history of *B. napus* breeding.

In addition, in order to compare the gene expression levels between the hot regions (regions with a CO frequency greater than 2 cM/Mb) and other regions (regions with a CO frequency less than 1 cM/Mb; regions with a CO frequency between 1 and 2 cM/Mb), we mapped all the genes to the reference genome of ZS11 (http://yanglab.hzau.edu.cn/BnIR/expression_zs11, accessed on 11 November 2022), and 51,584 genes were successfully mapped. A total of 11 tissues, such as roots, stems, leaves, seeds, and siliques, were selected for gene expression level analysis. In the petal and sepal, there were no significant differences in the average gene expression level in the different regions (Figure 2B, j–k). In the other nine tissues, the average gene expression level in the hot regions was significantly higher than that in the regions with a CO frequency of less than 1 cM/Mb (Figure 2B, a–i, Table S6 in the Supplementary Materials). These results suggested that the CO frequency of a region might be positively correlated with the expression level of its genes.

### 2.4. Homologous Genome Exchanges May Occur between the Subgenomes

**Figure 4:**
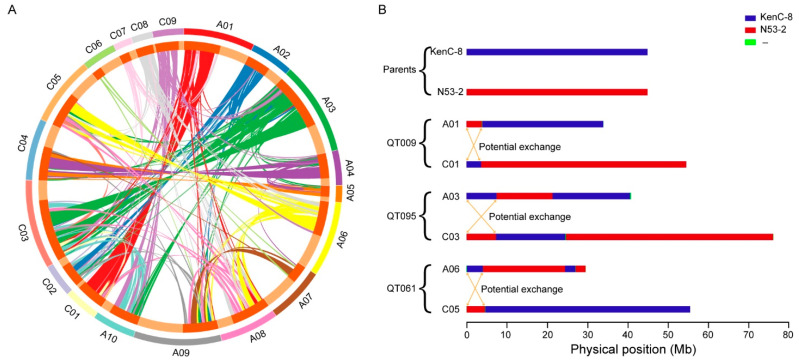
Potential exchanges between the chromosomes. (**A**) Homologous segment detection in the CO hot regions of the 19 chromosomes. Homologous segments were defined as those regions with more than 10 consecutive homologous genes, and the relationship between a pair of homologous and/or homologous genes is shown with a single line. The outer circle represents the chromosome name. In the inner circle, the orange-red bars represent the homologous genes, and the light tan bars represent the non-homologous regions. (**B**) Homologous genome exchanges may occur between the A and C subgenomes. Some lines, such as QT009, QT061, and QT095, may show homologous segment exchange in the CO hot regions.

To evaluate the homologous exchanges between the A and C subgenomes in the KN DH population, their homologous segments in the CO hot regions were compared. A homologous chromosomal segment was defined as one with more than 10 consecutive homologous gene pairs. As a result, a large number of homologous segments were detected across the whole *B. napus* genome (Figure 4A). Most putative exchangeable segments from the A subgenome of A01, A02, A03, and A04 were found, respectively, homologous to the C subgenome of C01, C02, C03, and C04. Further analysis showed that at the distal ends of the long arm of chromosome A03, a total of 943 genes (about half of the genes in this region) were homologous to the same position of chromosome C03. Meanwhile, we also found that lines QT095 and QT181 had a similar length to the exchanged segment in these regions (Figure 4B). These results implied that homologous genome exchanges might occur at the distal ends of the long arms of the A03 and C03 chromosomes. Similar phenomena were also observed between the chromosomes A01 and C01 of QT009, as well as between the chromosomes A06 and C05 of QT061 (Figure 4B). However, further experiments are required to consolidate these speculations. The homologous segments from the rest of the chromosomes were found to be in a diffuse distribution. Meanwhile, some exchangeable homologous segments were also found inside the A subgenome and inside the C subgenome (Figure 4A).

### 2.5. Bin Map Construction and Plant Phenotype Study

To assess the effectiveness of the potential bins associated with plant phenotypes, a bin map using 292 lines was constructed. A total of 1995 recombination bins out of the 2522 that were identified were successfully assigned to 19 linkage groups to construct the bin map (Table S7 in the Supplementary Materials). The total genetic size of the bin map was 1917.4 cM, with a length of 933.75 cM of the A subgenome and 983.65 cM of the C subgenome. The average distance between two adjacent bins was 0.96 cM.

**Figure 5:**
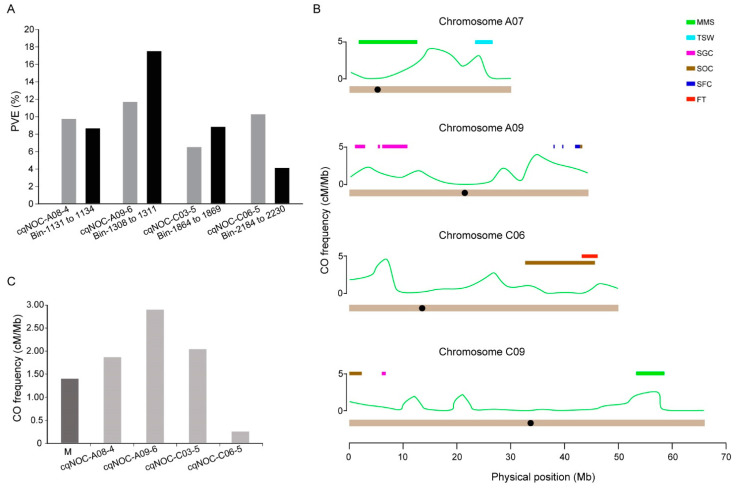
Bin map construction and the distribution of the QTL intervals of six plant phenotypes on the whole genome. (**A**) Four major oil-content QTL, as detected by the construction bin map. The black bars represent the four major QTL detected previously, and the gray bars represent the QTL detected in this study. (**B**) QTL for six traits mapped on the bin map. SOC, seed oil content; FT, flowering time; SGC, seed glucosinolate content; SFC, seed fiber components; MMS, multi-main stem; TSW, thousand-seed weight. (**C**) CO frequency in four major QTL intervals. The dark grey bar indicates the average CO frequency of the whole genome, and the light gray bars indicate the CO frequency in the four major QTL intervals, respectively.

SOC was one of the most important traits in rapeseed, and this trait was first used to verify the accuracy of the bin map. The potential loci controlling SOC were detected, based on the previously reported phenotypes collected in 10 environments by our group [26,27] (Figure S3 in the Supplementary Materials). It was found that the intervals of Bin 1131 to 1134, Bin 1308 to 1311, Bin 1864 to 1869, and Bin 2184 to 2230, from the chromosomes of A08, A09, C03, and C06, were tightly correlated to SOC. This could explain 8.5%, 17.3%, 8.6%, and 3.9% of the phenotypic variation, respectively. The four bin intervals shared almost the same physical intervals with the four major SOC quantitative trait loci (QTL) of *cqNOC-A08-4*, *cqNOC-A09-6*, *cqNOC-C03-5*, and *cqNOC-C06-4* that we identified in our previous research [26]. Further study showed that the phenotypic variations of the intervals of Bin 1131 to 1134, Bin 1308 to 1311, and Bin 1864 to 1869 were consistent with those of the three QTL of *cqNOC-A08-4*, *cqNOC-A09-6*, and *cqNOC-C03-5*. The interval of the QTL *cqNOC-C06-4* was more than twice as large as that of Bin 2184 to 2230 (Figure 5A). Overall, the bin map was accurate and could be used for the analysis of other traits.

Aside from the trait of SOC, QTL for other five traits including flowering time (FT), seed glucosinolate content (SGC), seed fiber components (SFC), multi-main stem (MMS), and thousand seed weight (TSW) in the KN DH population were also successfully mapped on the bin map (Figure 5B and Figure S4 in the Supplementary Materials). Most of the QTL for these traits tended to be distributed far away from the centromere, while the MMS QTL on chromosome A07 across the centromere were rare. SFC and SOC QTL were linked to a whole interval on chromosome A09, and there was a common interval of SOC and FT QTL on chromosome C06.

At the whole-genome level, QTL for the same trait had different CO frequencies in their different intervals. For example, among the four SOC QTL, the CO frequencies of the *cqNOC-A08-4*, *cqNOC-A09-6*, and *cqNOC-C03-5* intervals were 1.83 cM/Mb, 2.85 cM/Mb, and 2.00 cM/Mb, respectively, which values were significantly higher than the average values of the whole genome (Figure 5C), but the CO frequency of the *cqNOC-C06-5* interval was extremely low (0.22 cM/Mb). The CO frequency of the QTL interval could be instructive for future study. The construction of segregating populations based on the QTL for these traits was an effective method by which to further identify the candidate genes, and the sizes of these segregating populations were determined by their CO frequencies. For example, the CO frequency of the SOC QTL *cqNOC-A09-6* was 2.85 cM/Mb, suggesting that a medium-size segregating population could further narrow its interval; however, the CO frequency of the SOC QTL *cqNOC-C06-5* was 0.22 cM/Mb, thus indicating that an identification of the candidate genes of the trait required the construction of a large-scale segregating population. Together, these results deepened our understanding of the relationship between meiotic recombination and plant phenotypes at the population level.

## 3. Discussion

Recombination populations offer ideal models for studying plant phenotypes at the genomic level, where the potential loci associated with intriguing traits and the underlying mechanisms could be identified and illuminated [28]. COs are caused by the generation of DSBs, but only a minority of DSBs can be eventually repaired as COs; most of the rest finally become NCOs. In plants, only around 5% of the DSBs can turn to COs; this suggests that CO formation is strictly regulated and selected [29,30]. CO numbers show discrepancies in different organisms. For example, humans have about 2.6 COs per chromosome [31], while there are only 1.2 COs per chromosome in mice [32].

In plants, the number of COs in one chromosome is generally one to three [8]; approximately 1.6 and 2 COs were found in maize and *A. thaliana*, respectively [19,33]. The present results showed that there was an average of 23 COs in each line, and the COs mostly occurred at the distal ends of each chromosome. They were detected with an average of 1.2 COs per chromosome in one KN DH line, which was comparable to those reported in previous studies [8]. The number of COs per chromosome was positively correlated with the chromosome length, and this finding was similar to those reported in other studies [11,18,34,35]. However, the correlation was not as strong as that unveiled before (R = 0.483), thus indicating that chromosome length was not the only factor that affected the COs. Sex differences also could lead to the divergence of CO numbers. In humans, the number of COs in females was 1.6 times that in males [36]; however, the opposite results were found in maize, where the CO number in male parts was greater than that in female parts [19]. Selfish genetic elements might also alter CO production patterns. In rice, one such selfish genetic element disobeyed Mendel’s laws of inheritance, preferentially transmitting DNA segments from one parent to its offspring by altering the pattern of CO formation [37]. Except for rice, selfish elements were also found in maize [38]. Environmental conditions could also affect CO production; for instance, the number of COs was found to increase with the elevating temperature [39]. The number of COs per chromosome of each line was distinct in the KN DH population, which might be explained by complex factors, especially environmental conditions.

Generally, gene density gradually increases from centromeres to telomeres [40]. In the present study, most of the CO hot regions occurred at the distal ends of chromosomes and harbored a higher proportion of genes than those of their intervals. Quite a few genes in these regions were annotated with the functions of plant defense and regulation. For example, the most significant subcategory—the regulation of the salicylic acid-mediated signaling pathway—played a key role in the host response to biotrophics and pathogens [41,42]. At the same time, it was revealed that the A subgenome had more defense genes than those of the C subgenome, which finding was consistent with previous studies [22,43], as was consistent with the present results. This difference might be caused by the high-intensity natural and artificial selection exerted on the A subgenome in breeding history. In contrast, CO frequency was extremely low in the peri-centromere regions, even though these regions contained a considerable number of genes [40,44]. The low CO frequency made these regions a major challenge for breeders to obtain enough genetic variations [45].

Compared to the diploid species, polyploidy organisms typically harbored more COs with the same genetic background [46,47]. Similarly, the majority of COs in the allotetraploid *B. napus* were produced between the homologous chromosomes [48], and only a small proportion of them was detected between their homologous chromosomes [49]. Low-proportion bivalents could be formed between the A and C subgenome chromosomes at the zygotene stage and eventually induced recombination exchanges [50], while homologous exchanges usually occurred at the ends of the chromosomes [51]. In this study, the exchanges of some homologous fragments between the A and C subgenomes were also observed. For example, the DH lines of QT009, QT061, and QT095 had similar substitution lengths in the homologous regions between the A and C subgenomes.

Compared to the single-cell sequencing used for detecting the CO landscape [18,19], here, we present a cost-effective and informative strategy to analyze most of the CO patterns and their relationships with the more intriguing phenotypes at the population level. We mapped the QTL intervals of six traits to the bin map, and their intervals usually tended to be distributed far away from the centromere. For the same trait, the CO frequency of the QTL interval was different, which was instructive for a specific QTL to further construct a suitable segregating population by which to identify the candidate genes. This method provides a good reference for other species to study the CO distribution. Our study provides insights into understanding the recombination patterns in *B. napus*. However, the molecular mechanism underlying the formation of COs needs to be further analyzed.

## 4. Materials and Methods

### 4.1. Plant Materials and DNA Extraction

The KN DH population was derived from the microspore culture of the F_1_ hybrid between the parent plants, KenC-8 and N53-2 [24]. This population was planted in Wuhan, Hubei Province, in 2014, and their genomic DNA was extracted from the young floral buds using the CTAB method [52].

### 4.2. Genotyping and SNP Identification

The KN DH population was genotyped using the *Brassica* 60K array (Illumina Inc., USA) following the manufacturer’s protocol [53]. The genotyping data were scanned and exported by the Genome Studio software (Illumina Inc., USA). All SNP probe sequences were subject to BLAST analysis against the ZS11 reference genome [25]. Subsequently, SNP probe sequences were screened with the following criteria: (1) filter out the sequences with lengths of less than 50 bp; (2) only include the cases where the sequence identification values were 100%; (3) remove uncertain chromosome SNPs; (4) polymorphic SNPs were used for genotyping.

### 4.3. Bin and CO Validation

The consecutive SNPs showed an identical genotype across the whole population that was regarded as a bin. An in-house Python script was used to classify the bins, and the generated bins were manually corrected to avoid errors. Ambiguous SNPs were assigned to their closest bins. The junction of two adjacent bins was defined as a CO. The CO frequency (cM/Mb) was defined as the genetic distance (cM) versus the physical distance (Mb) [54]. CO hot regions were designated as the regions with a CO frequency greater than 2 cM/Mb.

### 4.4. Gene Analysis of the CO Hot Regions

Locations of the CO hot regions were determined by the ZS11 reference genome [25], and the associated genes were searched using the reference genome of Darmor-*bzh* [22]. Identification of the homologous genes was based on the *Arabidopsis thaliana* database (https://www.arabidopsis.org/, accessed on 17 September 2022). All the genes in the CO hot regions were enriched by gene ontology (GO) using Metascape [55]. The expression levels of all genes in the different tissues were retrieved from BnIR (http://yanglab.hzau.edu.cn/BnIR/expression_zs11, accessed on 11 November 2022).

### 4.5. Bin Map Construction and Plant Phenotype Analysis

Each bin was regarded as a molecular marker, and these markers were used to construct a linkage map via Joinmap 4.0 software with the Kosambi function [56]. The putative QTL were identified using the Windows QTL Cartographer 2.5 software with composite interval mapping [57], following the parameters of 10-cM of the scan window size, 2-cM of the computed interval, and a value of 2.5 of the LOD threshold. The bin map was used to detect the QTL associated with SOC. The SOC data was cited from our previous studies [26,27]. The QTL for FT [58], SGC [59], SFC [60], MMS [61], and TSW [62] were also obtained from our previous studies.

## 5. Conclusions

In this paper, we used a cost-effective, uncomplicated, and high-resolution method to study recombination frequency in the KN DH population. The results showed that there were 23 COs, on average, in each line, and these COs were unevenly distributed across the whole genome and mostly occurred at the distal ends of each chromosome. The genes found in the CO hot regions typically play a role in regulation and defense throughout the plant’s development process. This approach could be directly applied to other *Brassica* plants to detect the recombination frequency and provides a reference for their distant species.

## Data Availability

All data was enclosed in the main text and supplementary infor-mation files. Any detailed datasets generated during and/or analyzed during the current study are available from the corresponding author on reasonable request.

## Supplementary Materials

The following are available online at https://www.mdpi.com/article/10.3390/ijms24054469/s1.
