# Supplementary figures and images for: Dissecting the Meiotic Recombination Patterns in a Brassica napus Double Haploid Population Using 60K SNP Array
Source: Int J Mol Sci. 2023 Feb 24;24(5):4469. doi: 10.3390/ijms24054469 (PMC10003086; doi:10.3390/ijms24054469)

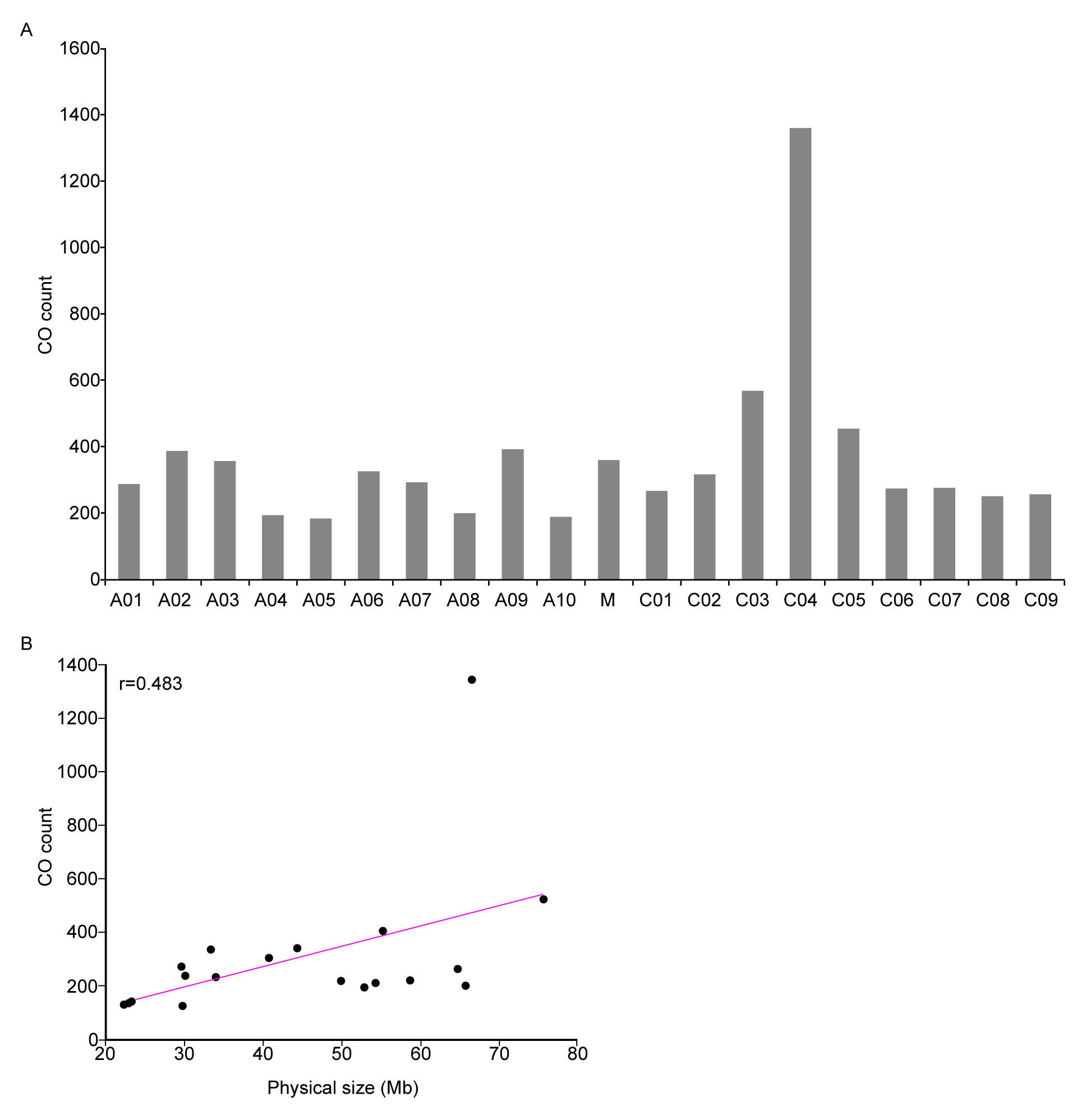

Supplement: Supplementary file 1 [file ijms-24-04469-s001.zip › Figure S1.jpg]

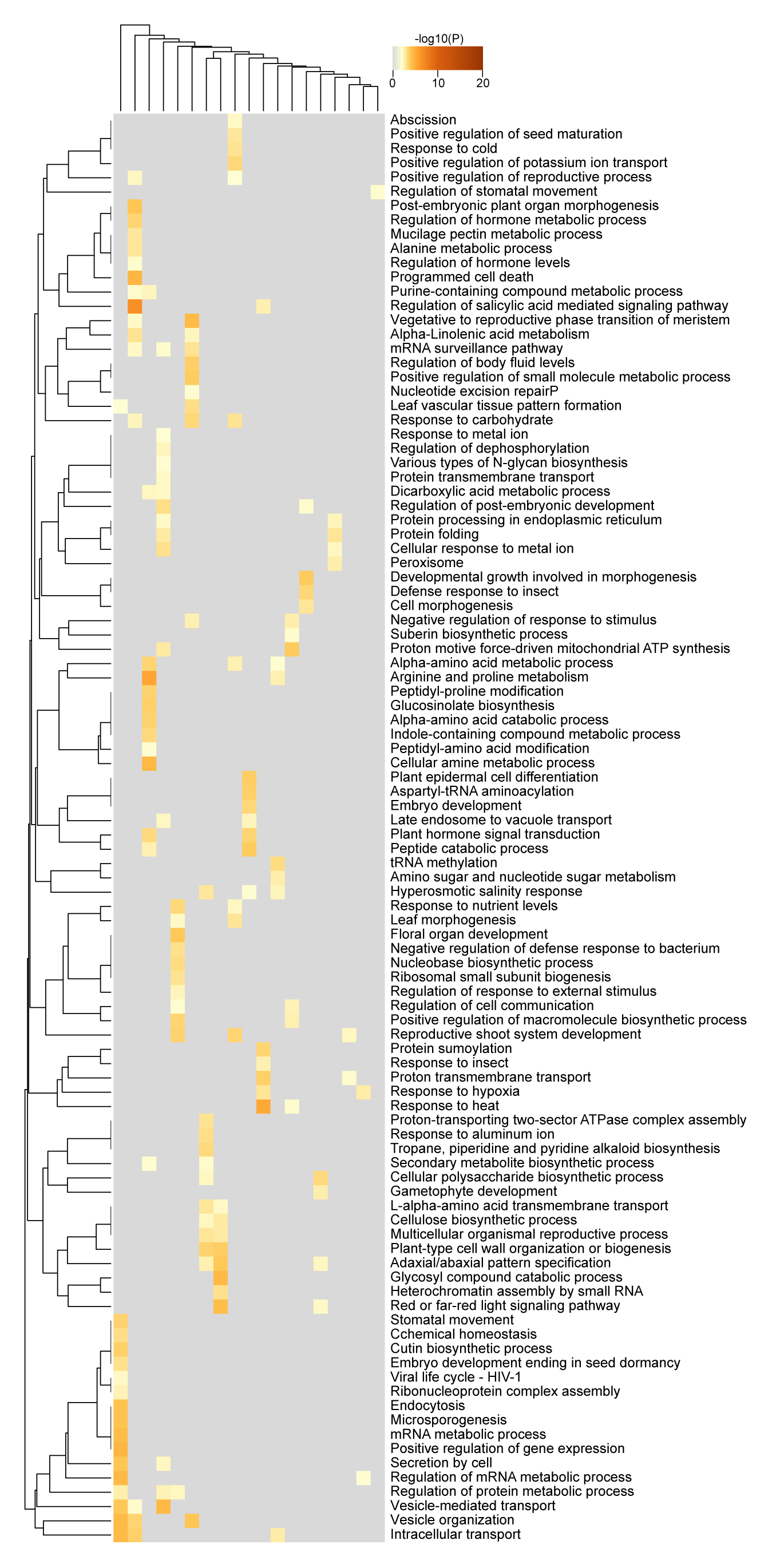

Supplement: Supplementary file 1 [file ijms-24-04469-s001.zip › Figure S2.jpg]

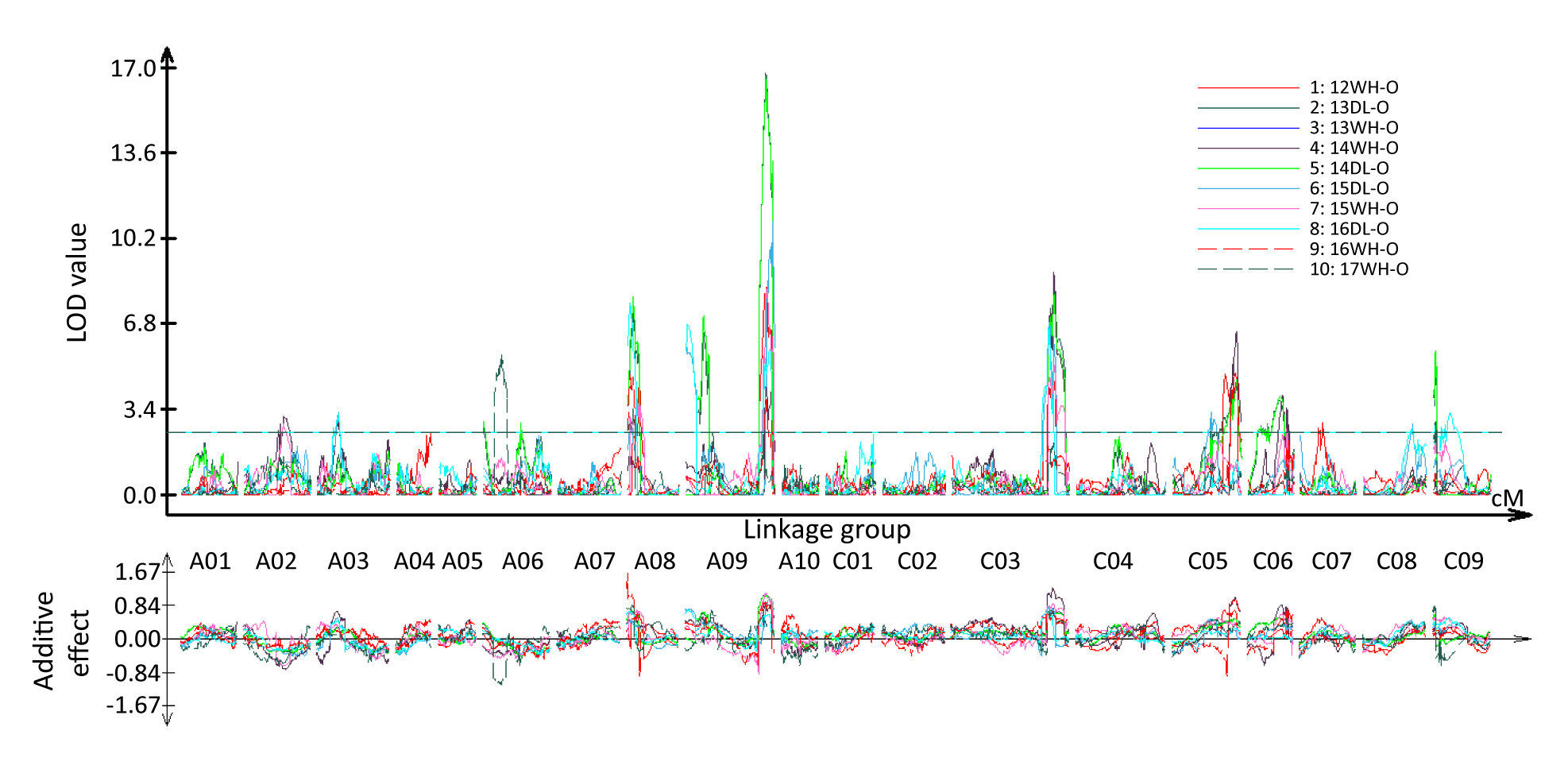

Supplement: Supplementary file 1 [file ijms-24-04469-s001.zip › Figure S3.jpg]

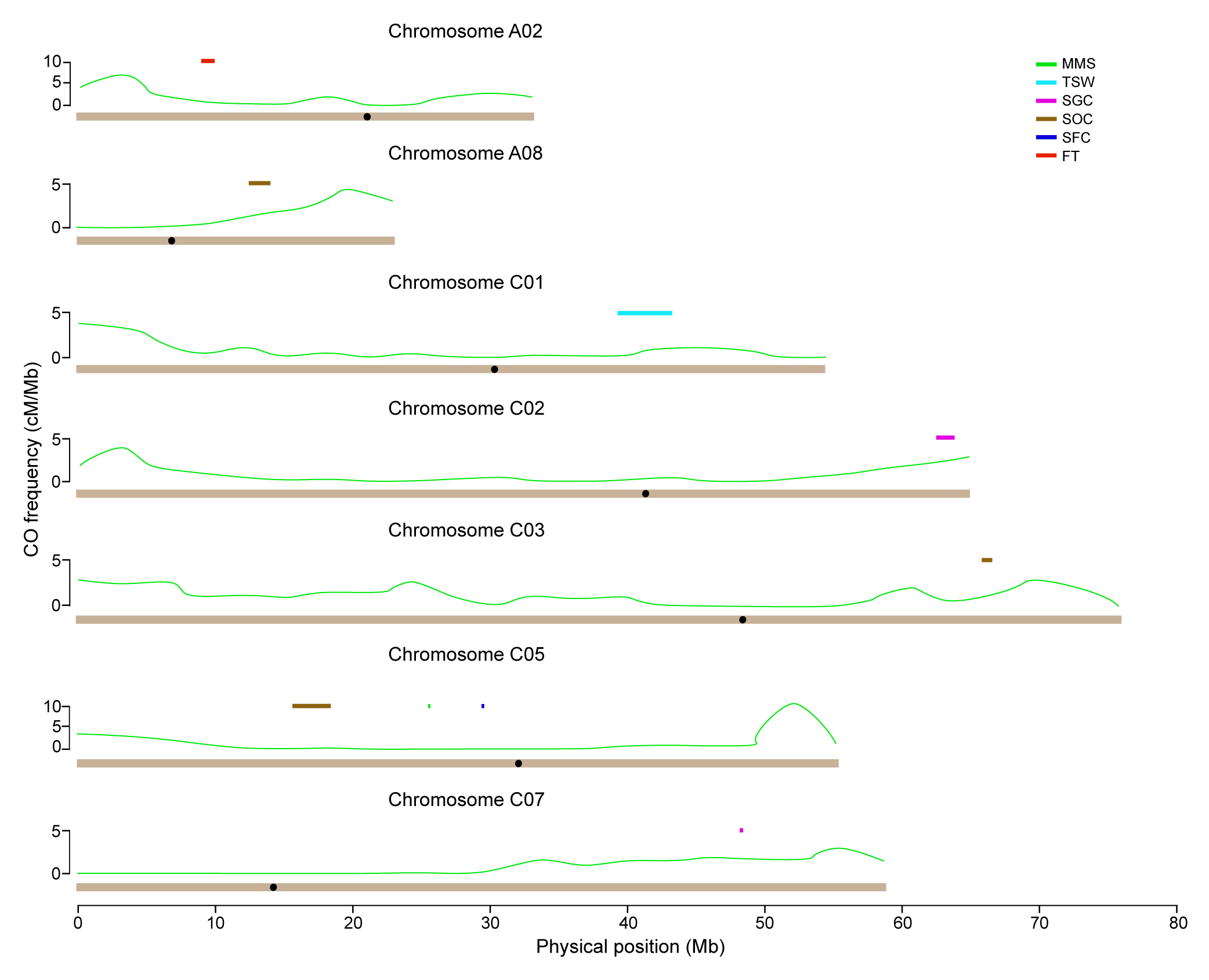

Supplement: Supplementary file 1 [file ijms-24-04469-s001.zip › Figure S4.jpg]
